# Efficacy of Acupuncture for Treating Opioid Use Disorder in Adults: A Systematic Review and Meta-Analysis

**DOI:** 10.1155/2018/3724708

**Published:** 2018-12-02

**Authors:** Zhihan Chen, Yitong Wang, Rui Wang, Jin Xie, Yulan Ren

**Affiliations:** ^1^School of Acupuncture Moxibustion and Tuina, Chengdu University of Traditional Chinese Medicine, Chengdu, Sichuan, China; ^2^School of Basic Medicine, Chengdu University of Traditional Chinese Medicine, Chengdu, Sichuan, China; ^3^School of Chinese Classics, Chengdu University of Traditional Chinese Medicine, Chengdu, Sichuan, China

## Abstract

**Objectives:**

To assess the efficacy of acupuncture in treating opioid use disorder (OUD).

**Design:**

Systematic review and meta-analysis.

**Methods:**

PubMed, Cochrane Central Register of Controlled Trials (CENTRAL), Embase, PsycINFO, Cumulative Index to Nursing and Allied Health Literature (CINAHL), Web of Science, ProQuest Dissertation and Theses, Allied and Complementary Medicine Database (AMED), Clinicaltrials.gov, and who.int/trialsearch were searched from inception to 23 December 2017. The methodological quality of selected studies and the quality of evidence for outcomes were assessed, respectively, by the Cochrane risk of bias assessment tool and the GRADE approach. Statistical analyses were conducted by RevMan 5.3.

**Results:**

A total of nine studies involving 1063 participants fulfilled the inclusion criteria. The results showed that acupuncture could be more beneficial than no treatment/sham acupuncture in terms of changes in craving for opioid (MD -2.18, 95% CI -3.10 to -1.26), insomnia (MD 2.31, 95% CI 1.97 to 2.65), and depression (SMD -1.50, 95% CI -1.85 to -1.15). In addition, these findings showed that, compared to sham electroacupuncture (EA), EA had differences in alleviating symptoms of craving (SMD -0.50, 95% CI -0.94 to -0.05) and depression (SMD -1.07, 95% CI -1.88 to -0.25) and compared to sham transcutaneous acupoint electrical stimulation (TEAS), TEAS had differences in alleviating symptoms of insomnia (MD 2.31, 95% CI 1.97 to 2.65) and anxiety (MD -1.26, 95% CI -1.60 to -0.92) compared to no treatment/sham TEAS.

**Conclusions:**

Acupuncture could be effective in treating OUD. Moreover, EA could effectively alleviate symptoms of craving for opioid and depression, and TEAS could be beneficial in improving symptoms of insomnia and anxiety. Nevertheless, the conclusions were limited due to the low-quality and small number of included studies. PROSPERO registration number is CRD42018085063.

## 1. Introduction

Opioid use disorder (OUD) is a serious substance-related disorder resulting from abuse or misuse of opioids [1]. The “World Drug Report 2017” [2] has declared that an estimated 250 million people used drugs at least once in 2015, around 29.5 million of those drug users, suffer from drug use disorders. With the increases in the prevalence of drug use disorders and the size of population, the disability-adjusted life-years (DALYs) attributed to drug use disorders increased by 24 percent from 2005 to 2015 [3, 4]. Currently, opioids remain the most harmful drug type in health terms, which cause 70 percent of the global burden of disease attributable to drug use disorders [2, 4]. Previous studies have shown that patients with OUD have a high risk of death and high rates of infectious diseases, for instance, human immunodeficiency virus (HIV), acquired immune deficiency syndrome (AIDS), and hepatitis B and C [5–9]. OUD is a worldwide health problem that seriously aggravates the burden on the individuals, family, and society [10, 11].

International guidelines recommend opioid substitution treatment (OST), namely, pharmacotherapy (buprenorphine, methadone, etc.); in addition, the primary clinical treatment for OUD also includes psychosocial treatment and acupuncture [10, 12–18]. Acupuncture has a long history in China, Japan, and Korea. With the development of the technique, acupuncture has become more varied [19]. At present, a growing number of countries have formulated regulations and policies for acupuncture [20]. As it is safe, is efficacious, and does not involve the ingestion of drugs, an increasing number of patients prefer to receive complementary and alternative treatments, such as acupuncture, to treat diseases. Previous clinical studies of the efficacy of acupuncture in OUD have come to different conclusions [21–24]. Four previous meta-analyses and systematic reviews discussed related questions; however, one [25] focused on the efficacy of acupuncture in the treatment of psychological symptoms associated with OUD, the second study [14] only evaluated the effectiveness of auricular acupuncture (AA), the third study [26] did not focus on the efficacy of various modes of acupuncture in OUD, and the last study [27] only included trials published in English before 2006. Thus, we conducted this study to assess the efficacy of various modes of acupuncture in OUD through separately comparing acupuncture with no treatment, sham acupuncture, and other therapies for OUD in adults.

## 2. Methods

This study was performed according to the Cochrane Handbook for Systematic Reviews of Interventions (version 5.1.0) [28] and the Preferred Reporting Items for Systematic Reviews and Meta-Analyses (PRISMA) guidelines [28]. In addition, the protocol of this study was registered in PROSPERO (ID: CRD42018085063).

### 2.1. Search Strategy and Inclusion Criteria

We searched ten online databases, namely, PubMed, Cochrane Central Register of Controlled Trials (CENTRAL), Embase, PsycINFO, Cumulative Index to Nursing and Allied Health Literature (CINAHL), Web of Science, ProQuest Dissertation and Theses, Allied and Complementary Medicine Database (AMED), Clinicaltrials.gov, and who.int/trialsearch, from inception to 23 December 2017, using search terms acupuncture, acupressure, point, opioid, heroin, morphine, and so on. There were no language restrictions. Special search strategies of the abovementioned databases are presented in online Appendix 1.

Trials were included if they met the following criteria:

1. Types of studies: clinical randomized controlled trials (RCTs) and quasi-RCTs except crossover trials and cluster RCTs.
2. Types of participants: considering actual clinical conditions [29], we included adult patients with primarily OUD, as defined by the diagnostic criteria in the Diagnostic and Statistical Manual of Mental Disorder (DSM) or the International Classification of Diseases (ICD) or other validated criteria or clinical assessment, and excluded pregnant women.
3. Types of interventions: experimental interventions included acupuncture therapies, and control interventions included no treatment or sham acupuncture or other therapies, such as psychosocial interventions, pharmacological interventions, and other conventional interventions.

### 2.2. Outcome Assessments

Primary outcomes are (1) intensity of withdrawal syndrome; (2) duration of treatment; (3) number of positive urine samples for opioids.

Secondary outcomes are (1) intensity of pain, anxiety, depression, insomnia and other associated symptoms; (2) retention of treatment; (3) nature and rate of adverse effect.

### 2.3. Data Collection and Analysis

All articles identified through searches of the online databases were imported to the EndNote X8 (Clarivate Analytics, Pennsylvania, the United States) software. Two review authors (ZC and YW) independently screened all articles by reading theirs titles and abstracts and excluded articles which do not meet inclusion criteria. Afterwards, the two authors independently reviewed full-text of articles. If there is any controversy during the screening, the third review author (YR) read relevant information and decided whether or not to include the article.

Two review authors (ZC and YW) independently extracted data to a self-designed data extraction form, which included authors, publication data, study design, sample size, characteristics of participants, history of drug use, interventions, and outcomes. Two other review authors (RW and JX) independently checked extracted data to enhance the accuracy. Afterwards, data were imported to RevMan 5.3 (Copenhagen: The Nordic Cochrane Centre, The Cochrane Collaboration) software.

The Cochrane risk of bias assessment tool [30] was adopted to assess included studies' selection bias, performance bias, detection bias, attrition bias, and reporting bias. Two review authors (ZC and YW) independently graded the risk of bias for included studies as the following: low risk, high risk, or unclear risk. If necessary, the third review author (YR) was consulted.

The GRADE approach [31] was adopted to assess the quality of evidence for outcomes in the following comparisons: acupuncture compared to no treatment, acupuncture compared to sham acupuncture, and acupuncture compared to medication. Outcomes of quality assessments in summary of findings tables were generated by GRADEprofiler (GRADEpro) 3.6.1 (Evidence Prime Inc., Ontario, Canada). GRADE approach results in a quality assessment of a body of evidence in one of following four grades: high, moderate, low, and very low.

RevMan 5.3 was used to analyse data. For dichotomous outcomes, results were reported as risk ratios (RRs) with 95% confidence intervals (CIs). It should be noted that if there were no events in both groups, the study was excluded from the meta-analysis [30]. For continuous data, when outcomes were measured by the same scale, results were reported as mean differences (MDs) with 95% CIs; when outcomes were measured by different scales, results were reported as standardized mean differences (SMDs) with 95% CIs. I^2^ statistic was used to assess heterogeneity. If I^2^ statistic was greater than 50%, random-effects model was used to perform data analyses, whereas fixed-effect model was used to perform data analyses, if I^2^ statistic was less than or equal to 50%. For multiple-intervention study, relevant intervention groups were combined into a single group by the formulae in the Cochrane Handbook for Systematic Reviews of Interventions [30]. In addition, characteristics of the multiple-intervention studies are presented by table. Considering acupuncture styles and stimulation modes have influence on clinical therapeutic efficacy, we conducted subgroup analyses on these factors to determine if there were sufficient data. Sensitivity analysis was used to explore other sources of heterogeneity caused by methodological quality or clinical differences. We planned to perform sensitivity analysis through excluding studies with high risk of bias from analysis. If there were sufficient numbers of studies (at least ten studies) included in meta-analysis, reporting bias was assessed by funnel plot [30, 32].

## 3. Results

### 3.1. Description of Included Studies

#### 3.1.1. Characteristics of Studies

**Figure 1:**
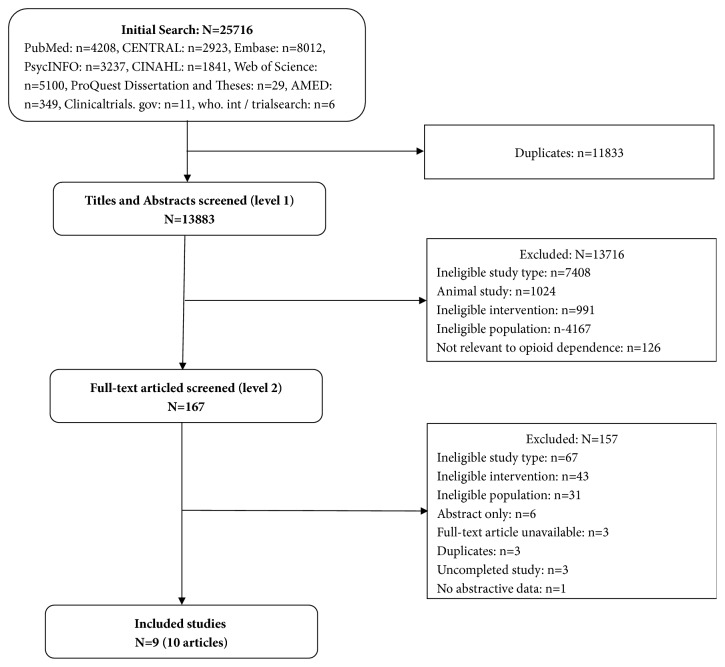
Flow chart of literature and screen process.

Nine studies [21–24, 33–38] with a total of 1063 participants were included in this study; thereinto, one study was reported by two articles [34, 35].  Figure 1 shows the process of selecting studies. A total of five studies [21–23, 33, 37] were published in English, and the others [24, 34–36, 38] were published in Chinese. All studies were reported by full-texts from 1993 to 2010, which were conducted in the United Kingdom [21], America [33, 37], and China [23, 24, 34–36] (two studies [22, 38] did not report the country in which studies are conducted). Among all studies, five studies [21, 24, 33, 37, 38] were two-armed trials, four studies [22, 23, 34–36] were four-armed trials, and the sample size ranged from 20 to 121 per arm. In included studies, all participants were diagnosed with OUD by DSM-IV [21, 23, 24, 34, 35], DSM-III-R [36], DSM-III [38], other validated criteria [34, 35], and clinical assessment [22, 33, 37]. There were differences in acupoint selection and stimulation modes. Manual acupuncture (MA) was used in 1 study [24], electroacupuncture (EA) was used in 4 studies [22, 23, 34–36], AA was used in 2 studies [21, 37], and transcutaneous acupoint electrical stimulation (TEAS) was used in 2 studies [33, 38]. Control group of all studies used no treatment [23, 34, 35], sham acupuncture [21, 23, 33–35, 37, 38], or medication [22, 24, 36]. Treatment lengths of included studies varied from four days to ten weeks, the total number of treatment sessions varied from ten to thirty, and each treatment session lasted from 20 to 45 minutes. In the aspects of outcome measures, 6 studies [21, 22, 24, 33–36] adopted different approaches to measure withdrawal syndromes (see Table 1), 3 studies [23, 24, 34, 35] reported the duration of treatment, 2 studies [36, 37] collected urine samples from participants for urine examination, 4 studies [21, 33–35, 38] adopted different approaches to measure craving for opioid, 2 studies [33, 38] used different scales to measure pain, 4 studies [23, 24, 34, 35, 38] used different scales to measure anxiety, 2 studies [23, 34, 35] used different scales to measure depression, 2 studies [33, 38] used different approaches to measure sleep, 5 studies [22–24, 33–35] reported retention, and no study reported adverse events.  Table 2 shows detailed characteristics of all included studies.

#### 3.1.2. Risk Bias in Included Studies

All included studies were described as RCTs. However, in random sequence generation, 1 study [21] used random number table, 2 studies [24, 33] used random sequence which was generated by computer, 1 study [34, 35] used envelopes, and 5 studies [22, 23, 36–38] did not report approach of random sequence generation. The approach of allocation concealment was assessed as low risk in 1 study [33], and the other [21–24, 34–38] studies did not report related information. In blinding, no study reported blinding of participants and outcome assessors, one study [37] did not report blinding but its outcome measurements were unlikely to be influenced by the lack of blinding, and the others [21–24, 33–36, 38] did not report method of blinding or did not provide enough information to permit judgement of “high risk of bias” or “low risk of bias”. In incomplete outcome data, 5 studies [23, 24, 33–35, 37] were assessed as “low risk of bias”; 3 studies [21, 36, 38] did not clearly report dropout rate or reasons for missing data; dropout rate of one study [22] was more than 20% and did not report reasons. In selective reporting, 1 study [33] was assessed as “low risk of bias”, 7 studies [22–24, 34–38] were assessed as “unclear risk of bias” due to no available study protocols, and 1 study [21] did not completely report all outcomes. In other bias, 6 studies [21, 22, 24, 33, 37, 38] were judged to be at “low risk of bias” and 3 studies [23, 34–36] were rated as being at “unclear risk of bias” due to insufficient information to permit judgement.

### 3.2. Effects of Intervention

Summaries of findings for all comparisons and GRADE analyses are presented in Tables 3, 4, and 5.

#### 3.2.1. Acupuncture versus no Treatment

*Intensity of Withdrawal Syndrome*. Considering different approaches of assessment and different ways of presenting the data, meta-analysis for the outcomes was limited [39, 40]. We attempted to summarize the outcomes in all comparisons. The details of scales are shown in Table 1. Mu et al., 2010 [34, 35], used withdrawal symptoms rating scale created by Liu Chuang to assess the outcome and showed statistical differences between EA and no treatment in the fourth week, eighth week, and tenth week.

*Duration of Treatment*. Two studies [23, 34, 35] reported the duration of treatment. However, the duration of treatment of the studies was set as 10 weeks, rather than determined by completion of treatment, and all participants in these studies completed treatment.

**Figure 2:**
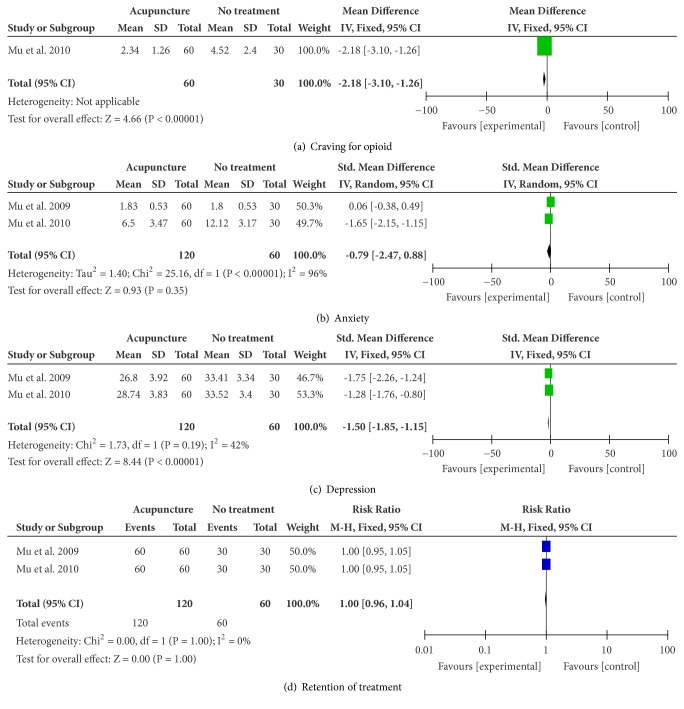
Meta-analyses of the effects of acupuncture compared to no treatment in treating OUD.

*Craving for Opioid*. One study [34, 35] reported the difference of craving for opioid between EA and no treatment, and EA significantly reduced craving for opioid (n = 90; MD, -2.18; 95% CI -3.10 to -1.26; p < 0.00001; Figure 2(a)).

*Anxiety*. The combined result showed no difference between EA and no treatment in reducing the severity of anxiety (n = 180; SMD, -0.79; 95% CI -2.47 to 0.88; p = 0.35; heterogeneity: X^2^ = 25.16, p < 0.00001, I^2^ = 96%; Figure 2(b)).

*Depression*. There was significant difference in depression between EA and no treatment (n = 180; SMD, -1.50; 95% CI -1.85 to -1.15; p < 0.00001; heterogeneity: X^2^ = 1.73, p = 0.19, I^2^ = 42%; Figure 2(c)).

*Retention of Treatment*. The combined result showed that there was no significant difference in retention (n = 180; RR, 1.00; 95% CI 0.96 to 1.04; p = 1.00; heterogeneity: X^2^ = 0.00, p = 1.00, I^2^ = 0%; Figure 2(d)).

*Nature and Rate of Adverse Effect*. No study reported adverse event.

#### 3.2.2. Acupuncture versus Sham Acupuncture

*Intensity of Withdrawal Syndrome*. The details of the scales are presented in Table 1. Mu et al., 2010 [34, 35], used withdrawal symptoms rating scale created by Liu Chuang to assess the outcome and showed statistical differences between EA and no treatment/sham acupuncture in the fourth week, eighth week, and tenth week. Bearn et al., 2009 [21], assessed intensity of withdrawal syndrome using Short Opiate Withdrawal Scale and showed no statistically significant differences between AA and sham AA on any of fourteen days. Meade et al., 2010 [33], adopted Subjective Opiate Withdrawal Scale to assess intensity of withdrawal syndrome at baseline, discharge, 1-week follow-up, and 2-week follow-up and showed no statistically significant difference between TEAS and sham TEAS.

*Duration of Treatment*. Two studies [23, 34, 35] reported the duration of treatment. All participants completed 10-week treatment.

**Figure 3:**
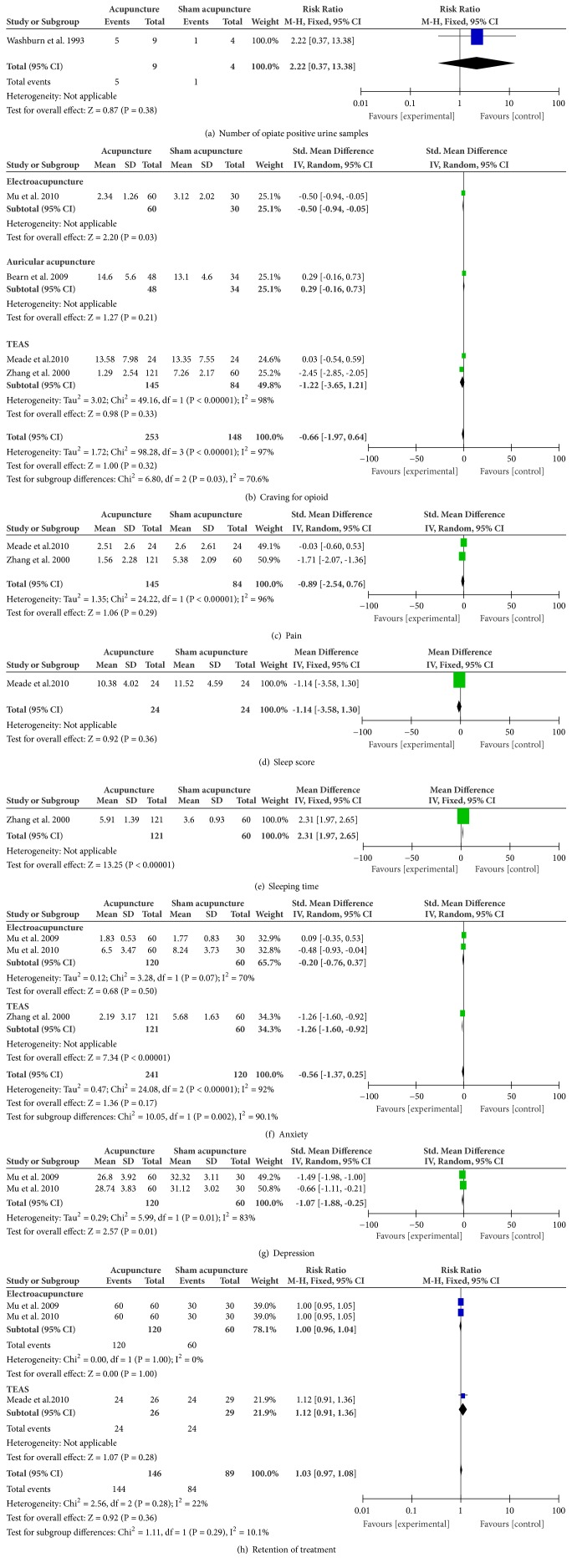
Meta-analyses of the effects of acupuncture compared to sham acupuncture in treating OUD.

*Number of Positive Urine Samples for Opioids*. Washburn et al., 1993 [37], reported that one participants was treated by sham AA and five participants were treated by AA tested positive for opioid. Meta-analysis showed that there was no significant difference in number of positive urine samples for opioids between AA and sham AA (n = 13; RR, 2.22; 95% CI 0.37 to 13.38; p = 0.38; Figure 3(a)).

*Craving for Opioid*. Acupuncture group and sham acupuncture group had no statistical difference in craving scores (n = 401; SMD, -0.66; 95% CI -1.97 to 0.64; p = 0.32; heterogeneity: X^2^ = 98.28, p < 0.00001, I^2^ = 97%; Figure 3(b)). Only one study was assessed as “high risk of bias”. Removing the study from the meta-analysis did not reduce heterogeneity (n = 319; SMD, -0.98; 95% CI -2.51 to 0.55; p = 0.21; heterogeneity: X^2^ = 65.07, p < 0.00001, I^2^ = 97%). In subgroup analyses, EA group and sham EA group had statistically significant difference in craving scores (n = 90; SMD, -0.50; 95% CI -0.94 to -0.05; p = 0.03; Figure 3(b)); AA group and sham AA group had no statistically significant difference in craving scores (n = 82; SMD, 0.29; 95% CI -0.16 to 0.73; p = 0.21; Figure 3(b)); TEAS group and sham TEAS group had no statistically significant difference in craving scores (n = 229; SMD, -1.22; 95% CI -3.65 to 1.21; p = 0.33; heterogeneity: X^2^ = 49.16, p < 0.00001, I^2^ = 98%; Figure 3(b)).

*Pain*. TEAS did not significantly relieve pain compared with sham TEAS (n = 229; SMD, -0.89; 95% CI -2.54 to 0.76; p = 0.29; heterogeneity: X^2^ = 24.22, p < 0.00001, I^2^ = 96%; Figure 3(c)).

*Sleep*. TEAS group and sham TEAS group had no statistically significant difference in sleep score (n = 48; MD, -1.14; 95% CI -3.58 to 1.30; p = 0.36; Figure 3(d)); however, TEAS group and sham TEAS group had statistically significant difference in sleeping time (n = 181; MD, 2.31; 95% CI 1.97 to 2.65; p < 0.00001; Figure 3(e)).

*Anxiety*. The combined result showed that acupuncture did not significantly reduce anxiety symptoms compared with sham acupuncture (n = 361; SMD, -0.56; 95% CI -1.37 to 0.25; p = 0.17; heterogeneity: X^2^ = 24.08, p < 0.00001, I^2^ = 92%; Figure 3(f)). No study used a “high risk of bias” method. In subgroup analyses, EA did not significantly reduce anxiety symptoms compared with sham EA (n = 180; SMD, 0.20; 95% CI -0.76 to 0.37; p = 0.50; heterogeneity: X^2^ = 3.28, p = 0.07, I^2^ = 70%; Figure 3(f)) and TEAS significantly reduced anxiety symptoms compared with sham TEAS (n = 181; MD, -1.26; 95% CI -1.60 to -0.92; p < 0.00001; Figure 3(f)).

*Depression*. There was statistical difference in depression score between EA and sham EA (n = 180; SMD, -1.07; 95% CI -1.88 to -0.25; p = 0.01; heterogeneity: X^2^ = 5.99, p = 0.01, I^2^ = 83%; Figure 3(g)).

*Retention of Treatment*. Low-quality evidence suggested that acupuncture group had no statistical difference in retention compared to sham acupuncture (n = 235; RR, 1.03; 95% CI 0.97 to 1.08; heterogeneity: X^2^ = 2.56, p = 0.28, I^2^ = 22%; Figure 3(h)). In subgroup analyses, there was no statistical difference in retention between EA and sham EA (n = 180; RR, 1.00; 95% CI 0.96 to 1.04; p = 1.00; heterogeneity: X^2^ = 0.00, p = 1.00, I^2^ = 0%; Figure 3(h)), and there was no statistical difference in retention between TEAS and sham TEAS (n = 48; RR, 1.12; 95% CI 0.91 to 1.36; p = 0.28; Figure 3(h)).

*Nature and Rate of Adverse Effect*. No study reported adverse events.

#### 3.2.3. Acupuncture versus Medication

*Intensity of Withdrawal Syndrome*. Hu et al., 2003 [22], used Himmelsbach scoring table for withdrawal symptoms and showed the results as a graph only. In the study, there was no significant difference in withdrawal syndromes score between EA and medication. Wen et al., 2005 [24], adopted score of abstinence symptoms to assess intensity of withdrawal syndrome and proposed that MA can reduce withdrawal syndromes on the fourth and fifth days. However, in the study, there were no significant differences between MA and medication when the treatment was completed. Zong et al., 2001 [36], used Himmelsbach scoring table for withdrawal symptoms and showed there was statistical difference in withdrawal syndromes score between EA and medication in the third day but there was no statistical difference between EA and medication when the treatment was completed.

*Duration of Treatment*. Only one study [24] reported the duration of treatment. All participants in the study completed 10-day treatment.

*Number of Positive Urine Samples for Opioids*. Zong et al., 2001 [36], reported that 0 of 20 participants treated by EA and 0 of 51 participants treated by medication tested positive for opioid.

**Figure 4:**
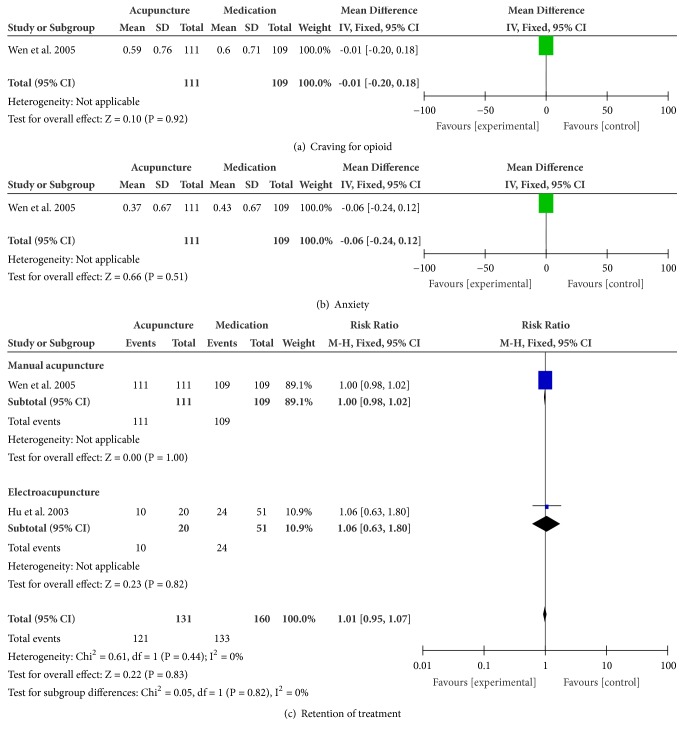
Meta-analyses of the effects of acupuncture compared to medication in treating OUD.

*Craving for Opioid*. There was no statistical difference between craving for opioid between MA and medication (n = 220; MD, -0.01; 95% CI -0.20 to 0.18; p = 0.92; Figure 4(a)).

*Anxiety*. There was no statistical difference in anxiety between MA and medication (n = 220; MD, -0.06; 95% CI -0.24 to 0.12; p = 0.51; Figure 4(b)).

*Retention of Treatment*. The combined result showed there was no statistical difference in retention between acupuncture and medication (n = 291; RR, 1.01; 95% CI 0.95 to 1.07; p = 0.83; heterogeneity: X^2^ = 0.61, p = 0.44, I^2^ = 0%; Figure 4(c)). In subgroup analyses, MA and medication have no statistically significant differences (n = 220; RR, 1.00; 95% CI 0.98 to 1.02; p = 1.00; Figure 4(c)); EA and medication also have no statistical difference (n = 71; RR, 1.06; 95% CI 0.63 to 1.80; p = 0.82; Figure 4(c)).

*Nature and Rate of Adverse Effect*. No study reported adverse events.

#### 3.2.4. Heterogeneity

In fact, acupuncture studies have high clinical heterogeneity owing to different acupuncture styles, different stimulation modes, different courses of acupuncture treatment, different acupuncture dosages, different choices of points, different context of acupuncture treatment, and so on. For EA, the duration was 20 days or 10 weeks, the frequency was three times per week or one time per day, and each treatment session lasted 20 minutes; for AA, the duration was 14 days or 21 days, the frequency was one time per day, and each treatment session lasted from 20 to 45 minutes; for TEAS, the duration was 4 days or 15 days, the total course of treatment was 12 sessions or 27 sessions, and each treatment session lasted 30 minutes. Point selections of all studies were not identical. Sham acupuncture studies also have clinical heterogeneity due to different stimulation modes and different stimulate positions. Medication controlled studies have heterogeneity on accounts of different types of drugs and different drug dosages. Because the number of studies was too small, we did not conduct these subgroup analyses. It was proposed to conduct sensitivity analyses through excluding studies with “high risk of bias”; however, sensitivity analyses for most meta-analyses were not performed because most studies did not provide necessary data and the amounts of studies were small. We conducted one sensitivity analysis for comparison between acupuncture and sham acupuncture in craving for opioid. And there was no significant change in craving for opioid after the removal of one study with “high risk of bias”.

#### 3.2.5. Reporting Bias

Owing to an insufficient number of included studies, we did not conduct analysis of reporting bias by funnel plot.

## 4. Discussion

The objective of this study is to assess the efficacy of various modes of acupuncture in OUD through intensity of withdrawal syndrome; duration of treatment; urine examination; intensity of pain, anxiety, depression, insomnia, and other associated symptoms; retention of treatment; and nature and rate of adverse effect. This study included 9 studies involving 1063 participants. There was certain difference between acupuncture and comparators, namely, no treatment, sham acupuncture, and medication, in treating OUD.

For intensity of withdrawal syndrome, it is impossible to arrive at a firm conclusion, due to the small number and low quality of studies. Nevertheless, the included studies indicated that EA and MA are effective for treating OUD. Data showed that EA was more effective in alleviating withdrawal syndromes than no treatment/sham acupuncture [34, 35], even medication on day 3 [36]; MA was more effective in alleviating withdrawal syndromes than medication on days 4 and 5 [24]. No data provided evidence of beneficial effects of AA and TEAS in treating OUD. For duration of treatment, it was set as a fixed duration (10 days or 10 weeks) before treatment in all studies, and all participants completed scheduled course of treatment. For urine examination, we found no evidence of the beneficial effects of AA in reducing number of positive urine samples for opioids, and the other one presented no participant was tested positive for opioid after being treated by EA or medication. In general, EA and MA could effectively relieve withdrawal syndromes, especially in short term; however, the low quality of studies limit our confidence of EA and MA in OUD.

Regarding secondary outcomes, EA could be effective in reducing craving for opioid and depression; TEAS could not improve sleep quality but could be effective in prolonging sleeping time; moreover, it was effective in reducing anxiety syndromes. The levels of evidence were very low to moderate, and most were very low to low. In addition, no data on adverse effects were available in all selected studies.

Although acupuncture could effectively treat OUD, considering of small sample sizes and low-quality studies, our findings warrant further high-quality studies with large samples size. Most studies were conducted in China, and the others were carried out in Kingdom and America. Acupuncture is applied in different countries through variable manners [41], and all included studies adopted various acupuncture regimens. Due to the differences of professional backgrounds of acupuncture manipulators and lack of standardized acupuncture regimens, the applicability of acupuncture in OUD was limited.

Overall, acupuncture could be effective in treating OUD, but the mechanism by which acupuncture alleviate OUD is not completely clear. The mechanism of acupuncture on OUD is probably related to opioid peptides, which are endogenous peptides with opiate-like activity. The three major classes currently recognized are dynorphins, enkephalins, and endorphins. Dynorphins can effectively suppress heroin withdrawal [42], and Han et al. indicated that acupuncture can increase dynorphin A [43]. Acupuncture can also significantly release enkephalins and endorphins [43, 44]. Furthermore, the mechanism is also possibly related to cAMP-response element binding protein (a protein functions to integrate both calcium and cAMP signals) [45], dopamine (one of the catecholamine neurotransmitters in the brain) content in the nucleus accumbens (NAc) [46], brain-derived neurotrophic factor (a member of the nerve growth factor family of trophic factors) [47], c-Fos (a protein encoded by the c-fos gene) expression of the amygdala [48], and postsynaptic neuronal activity in the nucleus accumbens and the striatum [49].

This study has several limitations. First, both the quantity of selected studies and the sample sizes of most studies were small. Second, some studies were of poor quality. Twenty-two percent of selected studies were assessed as “high risk of bias” [21, 22]; fifty-six percent of included studies did not report random sequence generation [22, 23, 36–38]; eighty-nine percent of these studies did not describe allocation concealment or blinding of outcome assessment [21–24, 34–38]; all studies did not report blinding of participants and personnel or provided insufficient information to judge if the blinding could have been broken; forty-four percent of studies did not report the amount, nature, or handling of incomplete outcome data [21, 22, 36, 38]; eighty-nine percent of studies did not provide available study protocols or report all outcomes [21–24, 34–38]. Third, all studies were conducted in China, Kingdom, and America; thus, the applicability of acupuncture in OUD was limited. Fourth, notwithstanding the fact that we made attempts to minimize bias, we hardly confirmed that all negative findings were published and grey literatures were included in this study.

Four previous meta-analyses and systematic reviews reported the effect of acupuncture on the treatment of OUD [14, 26, 27]. Zhang et al. [25] just assessed the effect of acupuncture in treatment of psychological symptoms associated with OUD, and both studies agreed that acupuncture could be effective in improving anxiety and depression. Thanks to the differences in inclusion-exclusion criteria and subgroup analyses, there was disagreement about whether acupuncture could treat craving for opioid. First, because cocaine does not belong to opioids, we did not include participants with cocaine addiction; thus, we thought EA could significantly reduce craving for opioid compared to no treatment. Second, our study also indicated that acupuncture group and sham acupuncture group had no statistical difference in treating craving; however, subgroup analyses indicated that EA could be effective in reducing craving for opioid. Finally, both studies agreed there were no differences in improving craving between acupuncture and medication. Baker et al. [14] just assessed the effect of AA on OUD. Despite the differences in search dates and inclusion-exclusion criteria, both studies agreed that there was no conclusive evidence of the effect of AA in treating OUD. Grant et al. [26] presented there was no differences between acupuncture and comparators for substance use disorders (SUDs). Nevertheless, it included participants with alcohol, stimulants, and opioids substance use and its comparators included passive controls, sham acupuncture, treatment as usual, and active interventions. Jordan [27] showed that the evidence did not confirm that acupuncture is effective in treating OUD. However, the study only included trials published in English and did not assess the effect of various types of acupuncture on OUD.

## 5. Conclusion

In this systematic review and meta-analysis, acupuncture could be effective in treating OUD, but there was insufficient evidence to suggest better effect of acupuncture compared to medication. These findings also showed EA could be effective in alleviating symptoms of craving for opioid and depression, and TEAS could be effective in improving insomnia and anxiety; nevertheless, the findings were insufficient to support clinical use of AA in treating OUD. The safety of acupuncture therapy in treating OUD was uncertain. To be noted, these results of the effects of acupuncture for OUD are limited by small number and low quality of selected studies.

## Figures and Tables

**Table 1:** Details of withdrawal scales.

**Studies**	**Name of scale**	**n** ^**0**^ ** items**	**n** ^**0**^ ** scores**
Bearn et al. 2009 [21]	Short Opiate Withdrawal Scale	10	3
Hu et al. 2003 [22]	Himmelsbach scoring table for withdrawal symptoms	10	One score for yawning, tear shedding, running nose and sweating separately; three scores for pupil dilation, trembling, gooseflesh, anorexia; five scores for restlessness and worry.
Meade et al. 2010 [33]	Subjective Opiate Withdrawal Scale	21	4
Mu et al. 2010 [34, 35]	Withdrawal symptoms rating scale (created by Liu Chuang)	10	3
Wen et al. 2005 [24]	Score of abstinence symptoms	17	15
Zong et al. 2001 [36]	Himmelsbach scoring table for withdrawal symptoms	13	One score for yawning, tear shedding, running nose, sweating, heat vexation, hyperpnea, rise of systolic hypertension; three scores for pupil dilation, trembling, gooseflesh, anorexia; five scores for worry and emesis.

**Table 2:** Characteristics of included studies.

**Included studies**	**Country**	**Study type**	**Sample size**	**Age (mean)**	**Sex**	**Time of drug abuse (mean)**	**Daily opioids use (mean)**	**Diagnosis**	**Intervention (Sample size)**	**Duration**	**Needle retention duration**	**Context of acupuncture treatment**	**Main outcomes**
Bearn et al. 2009 [21]	United Kingdom	RCT	82	35.99 years	62 males and 20 females	Not mentioned	Not mentioned	DSM-IV	Auricular acupuncture (n = 48); sham auricular acupuncture (n = 34)	14 days (10 sessions)	30 to 40 minutes	Before 14s-days acupuncture treatment, participants received 10 to 14 days decremental methadone therapy.	Intensity of withdrawal syndrome and craving.
Hu et al. 2003 [22]	Not mentioned	RCT	96	30.4 years	44 males and 52 females	29.5 months	1.16 g	Clinical assessment	Electroacupuncture (n = 20); (n = 28); Chinese herbs (n = 23); Acupuncture & Chinese herbs (n = 25)	20 days (20 sessions)	20 minutes	Not mentioned	Intensity of withdrawal syndrome.
Meade et al. 2010 [33]	America	RCT	48	27.5 years	33 males and 15 females	Not mentioned	Not mentioned	Clinical assessment	TEAS (n = 24); Sham TEAS (n = 24)	4 days (12 sessions)	30 minutes	Not mentioned	Intensity of withdrawal syndrome, carving, pain severity and interference; sleep quality.
Mu et al. 2009 [23]	China	RCT	120	29.43 years	48 males and 72 females	4.78 years	1.81 g	DSM-IV	Acupuncture group 1: electroacupuncture (n = 30); acupuncture group 2: electroacupuncture (n = 30); sham electroacupuncture (n = 30); no treatment (n = 30)	10 weeks (30 sessions)	20 minutes	Did not receive antipsychotic drugs in the previous week	Intensity of anxiety and depression
Mu et al. 2010 [34, 35]	China	RCT	120	29.84 years	48 males and 72 females	4.72 years	1.78 g	DSM-IV and ICD-10	Acupuncture group 1: electroacupuncture (n = 30); acupuncture group 2: electroacupuncture (n = 30); sham electroacupuncture (n = 30); no treatment (n = 30)	10 weeks (30 sessions)	20 minutes	Did not receive any detoxification treatment in the previous 3 months	Intensity of withdrawal syndrome, anxiety, depression, and Craving.
Washburn et al. 1993 [37]	America	RCT	100	40.46 years	68 males and 32 females	16.8 years	Not mentioned	Clinical assessment	Auricular acupuncture (n = 55); sham auricular acupuncture (n = 45)	21 days	20 to 45 minutes	All Participants received counseling and discharge planning, and AIDS education	Urine examination
Wen et al. 2005 [24]	China	RCT	220	33.8 years	171 males and 49 females	20.13 months	0.83 g	DSM-IV	Manual acupuncture (n = 111); western medicine (n = 109)	10 days (10 sessions)	30 minutes	Did not receive any detoxification treatment in the previous 1 month	Intensity of withdrawal syndrome and anxiety
Zhang et al. 2000 [38]	Not mentioned	RCT	181	26.54 years	Not mentioned	3.27 years	1.74 g	DSM-III	TEAS (n = 121); sham TEAS (n = 60)	15 days (27 sessions)	Not mentioned	Not mentioned	Sleeping time; intensity of pain, anxiety, addiction.
Zong et al. 2001 [36]	China	RCT	96	30.4 years	44 males and 52 females	15 months	1.18 g	DSM-III-R	Electroacupuncture (n = 20); Chinese herbs (n=23); western medicine (n=28); acupuncture & Chinese herb (n = 25)	20 days (20 sessions)	20 minutes	Not mentioned	Intensity of withdrawal syndrome; urine examination.

**Table 3:** Summary of findings: acupuncture versus no treatment.

**Outcomes**	**Number of RCTs**	**Number of Participants**	**Relative effect (95**%** CI)**	**Quality of the evidence (GRADE)** ^**∗**^
Craving for opioid	1	90	MD -2.18 (-3.10 to -1.26)	⊕⊕ ⊝⊝ low
Anxiety	2	180	SMD -0.79 (-2.47 to 0.88)	⊕ ⊝⊝⊝ very low
Depression	2	180	SMD -1.50 (-1.85 to -1.15)	⊕⊕ ⊝⊝ low
Retention of treatment	2	180	RR 1.00 (0.96 to 1.04)	⊕⊕ ⊝⊝ low

^**∗**^GRADE Working Group grades of evidence.

High quality: further research is very unlikely to change our confidence in the estimate of effect.

Moderate quality: further research is likely to have an important impact on our confidence in the estimate of effect and may change the estimate.

Low quality: further research is very likely to have an important impact on our confidence in the estimate of effect and is likely to change the estimate.

Very low quality: we are very uncertain about the estimate.

**Table 4:** Summary of findings: acupuncture versus sham acupuncture.

**Outcomes**	**Number of RCTs**	**Number of Participants**	**Relative effect (95**%** CI)**	**Quality of the evidence (GRADE)** ^**∗**^
Number of positive urine samples for opioids	1	13	RR 2.22 (0.37 to 13.38)	⊕⊕⊕⊝ moderate
Craving for opioid	4	401	SMD -0.66 (-1.97 to 0.64)	⊕⊝⊝⊝ very low
Pain	2	229	SMD -0.89 (-2.54 to 0.76)	⊕⊝⊝⊝ very low
Sleep quality	1	48	MD -1.14 (-3.58 to 1.30)	⊕⊕⊕⊝ moderate
Sleeping time	1	181	MD 2.31 (1.97 to 2.65)	⊕⊕⊕⊝ moderate
Anxiety	3	361	SMD -0.56 (-1.37 to 0.25)	⊕⊝⊝⊝ very low
Depression	2	180	SMD -1.07 (-1.88 to -0.25)	⊕⊝⊝⊝ very low
Retention of treatment	3	235	RR 1.03 (0.97 to 1.08)	⊕⊕⊝⊝ low

^**∗**^GRADE Working Group grades of evidence.

High quality: further research is very unlikely to change our confidence in the estimate of effect.

Moderate quality: further research is likely to have an important impact on our confidence in the estimate of effect and may change the estimate.

Low quality: further research is very likely to have an important impact on our confidence in the estimate of effect and is likely to change the estimate.

Very low quality: we are very uncertain about the estimate.

**Table 5:** Summary of findings: acupuncture versus medication.

**Outcomes**	**Number of RCTs**	**Number of Participants**	**Relative effect (95**%** CI)**	**Quality of the evidence (GRADE)** ^**∗**^
Craving for opioid	1	220	MD -0.01 (-0.20 to 0.18)	⊕⊕⊕⊝ moderate
Anxiety	1	220	MD -0.06 (-0.24 to 0.12)	⊕⊕⊕⊝ moderate
Retention of treatment	2	291	RR 1.01 (0.95 to 1.07)	⊕⊕⊕⊝ moderate

^**∗**^GRADE Working Group grades of evidence.

High quality: further research is very unlikely to change our confidence in the estimate of effect.

Moderate quality: further research is likely to have an important impact on our confidence in the estimate of effect and may change the estimate.

Low quality: further research is very likely to have an important impact on our confidence in the estimate of effect and is likely to change the estimate.

Very low quality: we are very uncertain about the estimate.

## Data Availability

No additional data were available.

## Supplementary Materials

Supplementary Materials Appendices 1. Search strategy: special search strategies of ten online databases, namely, PubMed, Cochrane Central Register of Controlled Trials (CENTRAL), Embase, PsycINFO, Cumulative Index to Nursing and Allied Health Literature (CINAHL), Web of Science, ProQuest Dissertation and Theses, Allied and Complementary Medicine Database (AMED), Clinicaltrials.gov, and who.int/trialsearch. Appendices 2. PRISMA 2009 checklist: PRISMA 2009 is a guideline for reporting of systematic reviews, and we use the guideline to structure our manuscript.
