# Supplementary material for: Efficacy of Acupuncture for Treating Opioid Use Disorder in Adults: A Systematic Review and Meta-Analysis
Source: Evid Based Complement Alternat Med. 2018 Dec 2;2018:3724708. doi: 10.1155/2018/3724708 (PMC6304557; doi:10.1155/2018/3724708)
Supplement: Supplementary Materials — Appendices 1. Search strategy: special search strategies of ten online databases, namely, PubMed, Cochrane Central Register of Controlled Trials (CENTRAL), Embase, PsycINFO, Cumulative Index to Nursing and Allied Health Literature (CINAHL), Web of Science, ProQuest Dissertation and Theses, Allied and Complementary Medicine Database (AMED), Clinicaltrials.gov, and who.int/trialsearch. Appendices 2. PRISMA 2009 checklist: PRISMA 2009 is a guideline for reporting of systematic reviews, and we use the guideline to structure our manuscript. [file 3724708.f1.zip › 3724708.f1/Appendices 1. Search strategy_ECAM_2591755.pdf]

1 PubMed search strategy

#1 "acupuncture"[MeSH Terms] or "acupuncture therapy"[MeSH Terms] or "electroacupuncture" [MeSH Terms] or "acupressure" [MeSH Terms] (21830)
#2 acupuncture*[tw] or pharmacopuncture[tw] or electroacupuncture*[tw] or electro-acupuncture*[tw] or acupressure*[tw] or point*[tw] or acupoint*[tw] (867445)
#3 meridian*[tw] or jingluo[tw] or jing-luo[tw] or "jing luo"[tw] or "ching lo"[tw] (5274)
#4 needl*[tw] (143750)
#5 #1 or #2 or #3 or #4 (1005400)
#6 "Opioid-Related Disorders"[MeSH Terms] OR "Opiate Substitution Treatment"[MeSH Terms] OR "Heroin"[MeSH Terms] OR "Morphine"[MeSH Terms] (59453)
#7 opioid*[tw] or heroin*[tw] or morphine*[tw] or opium*[tw] or opiate*[tw] (152143)
#8 narcot*[tw] or detoxifi*[tw] or desintoxi*[tw] or disintoxi*[tw] or disintossi*[tw] (86594)
#9 #6 or #7 or #8 (210205)
#10 randomized controlled trial[pt] (449854)
#11 controlled clinical trial[pt] (537037)
#12 randomized[tiab] (429126)
#13 placebo[tiab] (190057)
#14 drug therapy[sh] (1978849)
#15 randomly[tiab] (282617)
#16 trial[tiab] (489061)
#17 groups[tiab] (1773039)
#18 #10 or #11 or #12 or #13 or #14 or #15 or #16 or #17 (4162110)
#19 animals[MeSH Terms] not (humans[MeSH Terms] and animals[MeSH Terms]) (4406436)
#20 #18 not #19 (3598541)
#21 #5 AND #9 AND #20 (4208)

2 CENTRAL search strategy

#1 MeSH descriptor: [Acupuncture] explode all trees (137)
#2 MeSH descriptor: [Acupuncture Therapy] explode all trees (3736)
#3 MeSH descriptor: [Electroacupuncture] explode all trees (645)
#4 MeSH descriptor: [Acupressure] explode all trees (271)
#5 acupuncture* or pharmacopuncture or electroacupuncture* or electro-acupuncture* or acupressure* or point* or acupoint* or meridian* or needl* (89145)
#6 jingluo or jing-luo or "jing luo" or "ching lo" (1)
#7 #1 or #2 or #3 or #4 or #5 or #6 (89164)

#8 MeSH descriptor: [Opioid-Related Disorders] explode all trees (1374)
#9 MeSH descriptor: [Opiate Substitution Treatment] explode all trees (220)
#10 MeSH descriptor: [Heroin] explode all trees (267)
#11 MeSH descriptor: [Morphine] explode all trees (3814)
#12 opioid* or heroin* or morphine* or opium* or opiate* (21562)
#13 narcot* or detoxifi* or desintoxi* or disintoxi* or disintossi* (7071)
#14 #8 or #9 or #10 or #11 or #12 or #13 (25130)
#15 #7 AND #14 (2923)

3 Embase search strategy

#1 'acupuncture'/exp (40970)
#2 'pharmacopuncture'/exp (36)
#3 'electroacupuncture'/exp (5472)
#4 'acupressure'/exp (1751)
#5 acupuncture*:ab,kw,ti OR pharmacopuncture:ab,kw,ti OR electroacupuncture*:ab,kw,ti OR 'electro acupuncture*':ab,kw,ti OR acupressure*:ab,kw,ti OR point*:ab,kw,ti OR acupoint*:ab,kw,ti OR meridian*:ab,kw,ti OR needl*:ab,kw,ti (1227167)
#6 jingluo:ab,kw,ti OR 'jing luo':ab,kw,ti OR 'jing-luo':ab,kw,ti OR 'ching lo':ab,kw,ti (36)
#7 #1 OR #2 OR #3 OR #4 OR #5 OR #6 (1236813)
#8 'opiate addiction'/exp (14782)
#9 'opiate substitution treatment'/exp (1535)
#10 'heroin dependence'/exp (8896)
#11 'morphine addiction'/exp (3169)
#12 opioid*:ab,kw,ti OR heroin*:ab,kw,ti OR morphine*:ab,kw,ti OR opium*:ab,kw,ti OR opiate*:ab,kw,ti (169101)
#13 narcot* OR detoxifi* OR desintoxi* OR disintoxi* OR disintossi*:ab,kw,ti (93056)
#14 #8 OR #9 OR #10 OR #11 OR #12 OR #13 (248616)
#15 'randomized controlled trial'/exp (477419)
#16 'controlled clinical trial'/exp (635353)
#17 'controlled study'/exp (5852434)
#18 'double blind procedure'/exp (144063)
#19 'single blind procedure'/exp (29949)
#20 'crossover procedure'/exp (53406)
#21 'placebo'/exp (315379)
#22 random* OR allocat* OR assign* OR blind* OR placebo* OR followup OR 'follow-up' OR crossover OR 'cross-over' OR factorial* OR volunteer* OR group* (782040)
#23 (single* OR double* OR treble* OR triple*) AND (blind* OR mask*) (294439)
#24 (control* OR compar* OR prospective* OR clinical) AND (trial OR study OR design) (12155512)

#25 #15 OR #16 OR #17 OR #18 OR #19 OR #20 OR #21 OR #22 OR #23 OR #24 (15008821)
#26 #7 AND #14 AND #25 AND [humans]/lim (8012)

4 PsycINFO (via EBSCO) search strategy

S1 MA acupuncture OR MA acupuncture therapy OR MA Acupuncture, ear OR MA electroacupuncture OR MA meridians OR MA acupuncture points OR MA acupressure (1078)
S2 acupuncture* OR pharmacopuncture OR electroacupuncture* OR electro-acupuncture* OR acupressure* OR point* OR acupoint* OR meridian* OR needl* (252775)
S3 jingluo OR jing-luo OR "jing luo" OR "Ching lo" (112)
S4 S1 OR S2 OR S3 (252879)
S5 MA opioid-related disorders OR MA heroin dependence OR MA morphine dependence OR MA opium dependence OR MA opiate substitution treatment OR MA heroin OR MA morphine (14379)
S6 opioid* OR heroin* OR morphine* OR opium* OR opiate* (40864)
S7 narcot* OR detoxifi* OR desintoxi* OR disintoxi* OR disintossi* (17340)
S8 S5 OR S6 OR S7 (48386)
S9 S4 AND S8 (3237)

5 CINAHL (via EBSCO) search strategy

S1 MH acupuncture OR MH acupuncture therapy OR MH Acupuncture, ear OR MH electroacupuncture OR MH meridians OR MH acupuncture points OR MH acupressure (10147)
S2 acupuncture* OR pharmacopuncture OR electroacupuncture* OR electro-acupuncture* OR acupressure* OR point* OR acupoint* OR meridian* OR needl* (118435)
S3 jingluo OR jing-luo OR "jing luo" OR "Ching lo" (5)
S4 S1 OR S2 OR S3 (118437)
S5 MH opioid-related disorders OR MH heroin dependence OR MH morphine dependence OR MH opium dependence OR MH opiate substitution treatment OR MH heroin OR MH morphine (5264)
S6 opioid* OR heroin* OR morphine* OR opium* OR opiate* (23097)
S7 narcot* OR detoxifi* OR desintoxi* OR disintoxi* OR disintossi* (12045)
S8 S5 OR S6 OR S7 (30202)
S9 S4 AND S8 (1841)

6 Web of Science search strategy

#1 TS=(acupuncture* OR electroacupuncture* OR "electro-acupuncture*" OR

pharmacopuncture OR acupressure* OR point* OR acupoint* OR meridian* OR needl* OR jingluo OR "jing-luo" OR "jing luo" OR "ching lo") (1818607)
#2 TS=(opioid* OR heroin* OR morphine* OR opium* OR opiate* OR narcot* OR detoxifi* OR desintoxi* OR disintoxi* OR disintossi*) (191595)
#3 #1 AND #2 (8833)
#4 TS=((single* OR double* OR treble* OR triple*) SAME (blind* or mask*)) (261586)
#5 TS=((control* OR compar* OR prospective* OR clinical) SAME (trial OR study OR design)) (6504537)
#6 TS=(random* OR allocat* OR assign* OR blind* OR placebo* OR followup OR "follow-up" OR crossover OR "cross-over" OR factorial* OR volunteer* OR group*) (6107497)
#7 #4 OR #5 OR #6 (10234684)
#8 TS=(animals NOT (humans AND animals)) (729393)
#9 #7 NOT #8 (9903728)
#10 #3 AND #9 (5100)

7 ProQuest Dissertation and Theses search strategy

S1 su(acupuncture) OR su(acupuncture therapy) OR su(acupuncture, ear) OR su(electroacupuncture) OR su(meridians) OR su(acupuncture points) (155)
S2 all(acupuncture*) OR all(pharmacopuncture) OR all(electroacupuncture*) OR all(electro-acupuncture*) OR all(acupressure*) OR all(point*) AND all(acupoint*) AND all(meridian*) AND all(needl*) (594)
S3 all(jingluo) OR all(jing-luo) OR all("jing luo") OR all("Ching lo") (1)
S4 S1 OR S2 OR S3 (624)
S5 su("opioid-related disorders") OR su("morphine dependence") OR su("opium dependence") OR su("heroin dependence") OR su("opiate substitution treatment") OR su(heroin) OR su(morphine) (437)
S6 all(opioid*) OR all(heroin*) OR all(morphine*) OR all(opium*) OR all(opiate*) (10317)
S7 all(narcot*) OR all(detoxifi*) OR all(desintoxi*) OR all(disintoxi*) OR all(disintossi*) (1417)
S8 S5 OR S6 OR S7 (11500)
S9 S4 AND S8 (29)

8 AMED (via OVID) search strategy

1 exp Acupuncture/ (3298)
2 exp acupuncture therapy/ or exp acupoints/ or exp acupressure/ or exp ear acupuncture/ or exp electroacupuncture/ or exp meridians/ or exp needling/ or exp scalp acupuncture/ (7738)
3 "acupuncture*".ti,hw,ab. (9685)

4 pharmacopuncture.ti,hw,ab. (15)
5 "electroacupuncture*".ti,hw,ab. (1046)
6 "electro-acupuncture*".ti,hw,ab. (202)
7 "acupressure*".ti,hw,ab. (391)
8 "point*".ti,hw,ab. (11016)
9 "acupoint*".ti,hw,ab. (1996)
10 "meridian*".ti,hw,ab. (672)
11 "needl*".ti,hw,ab. (1843)
12 jingluo.ti,hw,ab. (5)
13 jing-luo.ti,hw,ab. (4)
14 "jing luo".ti,hw,ab. (4)
15 "Ching lo".ti,hw,ab. (0)
16 1 or 2 or 3 or 4 or 5 or 6 or 7 or 8 or 9 or 10 or 11 or 12 or 13 or 14 or 15 (20180)
17 exp substance related disorders/ or exp "substance use disorders"/ or exp substance abuse/ or exp drug abuse/ or exp substance dependence/ or exp substance withdrawal syndrome/ (1403)
18 "opioid*".ti,hw,ab. (1314)
19 "heroin*".ti,hw,ab. (46)
20 "morphine*".ti,hw,ab. (648)
21 "opium*".ti,hw,ab. (79)
22 "opiate*".ti,hw,ab. (130)
23 "narcot*".ti,hw,ab. (349)
24 "detoxifi*".ti,hw,ab. (293)
25 "desintoxi*".ti,hw,ab. (1)
26 "disintoxi*".ti,hw,ab. (0)
27 "disintossi*".ti,hw,ab. (0)
28 17 or 18 or 19 or 20 or 21 or 22 or 23 or 24 or 25 or 26 or 27 (3529)
29 16 and 28 (349)

9 Clinicaltrials. gov search strategy

1 acupuncture and opioid (3)
2 acupuncture and opiate (2)
3 acupuncture and heroin (2)
4 acupuncture and morphine (5)
5 electroacupuncture and opioid (4)
6 electroacupuncture and opiate (3)
7 electroacupuncture and heroin (2)
8 electroacupuncture and morphine (0)
9 acupressure and opioid (0)
10 acupressure and opiate (1)
11 acupressure and heroin (1)
12 acupressure and morphine (0)
13 1 or 2 or 3 or 4 or 5 or 6 or 7 or 8 or 9 or 10 or 11 or 12 (11)

10 who. int / trialsearch search strategy

1 acupuncture and opioid (3)
2 acupuncture and opiate (2)
3 acupuncture and heroin (0)
4 acupuncture and morphine (1)
5 electroacupuncture and opioid (1)
6 electroacupuncture and opiate (1)
7 electroacupuncture and heroin (0)
8 electroacupuncture and morphine (0)
9 acupressure and opioid (0)
10 acupressure and opiate (1)
11 acupressure and heroin (0)
12 acupressure and morphine (0)
13 1 or 2 or 3 or 4 or 5 or 6 or 7 or 8 or 9 or 10 or 11 or 12 (6)
